# Special Issue “Microorganisms in Recycling and Valorization of Organic Waste for Sustainable Soil Health and Management”

**DOI:** 10.3390/microorganisms9081682

**Published:** 2021-08-08

**Authors:** José A. Siles, Mercedes García-Sánchez, María Gómez-Brandón

**Affiliations:** 1 Department of Plant and Microbial Biology, University of California at Berkeley, Berkeley, CA 94720, USA; josesimartos@gmail.com; 2 Department of Soil and Water Conservation and Waste Management, CEBAS-CSIC, 30100 Murcia, Spain; 3 Eco & Sols, INRAE, Institut Agro, Université de Montpellier, CIRAD, IRD, CEDEX 02, 34060 Montpellier, France; garcia.sanchez.mercedes@gmail.com; 4 Grupo de Ecoloxía Animal (GEA), Universidad de Vigo, 36310 Vigo, Spain

Anthropogenic activity generates huge amounts of solid organic wastes. Their incorrect handling may result in soil, water and air contamination. Therefore, finding strategies to reuse and treat organic wastes is needed. Their use as soil organic amendments has been proposed as an effective way of improving soil quality and fertility, while at the same time, protecting the environment. However, if not properly disposed of and treated, the use of raw wastes as soil inputs may lead to harmful environmental effects. In this context, the adoption of biological approaches, such as composting, vermicomposting or methanogenic processes offers the possibility of stabilizing the organic wastes before their use as soil amendments, achieving a dual purpose: environmental protection and production of amendments with improved characteristics. The role of microorganisms in these processes is crucial since they mediate the conversion of organic matter through a variety of chemical reactions. Thus, their study is fundamental, as is that of microbial communities in organically amended soils due to their usefulness as bio-indicators of soil quality. Altogether, it will help to reach integrative assessments on the suitability of waste transformation processes and promote sustainable agricultural practices.

The objective of this Special Issue on “Microorganisms in Recycling and Valorization of Organic Waste for Sustainable Soil Health and Management” was to provide a platform to researchers for sharing their new studies on the characterization of microorganisms mediating the transformation of organic wastes into appropriate soil amendments and on the impact of the application of stabilized wastes on soil microbial communities, nutrient cycling and plant yields. The present Special Issue comprises 7 research articles and 2 reviews, contributed by 47 authors.

Three articles were focused on the study of microbial communities during processes of waste transformation. Santos et al. [1] provided evidence about the efficient use of industrial alkaline wastes including CaO as chemical conditioners to sanitize sewage sludge by drastically reducing its pathogenic load. Thermal treatment assays were also tested as feasible alternatives to reach this goal. Jeong et al. [2] evaluated the effectiveness of the composting process on the inactivation of avian influenza virus (H9N2) and the encephalomyocarditis virus (EMCV) present in poultry and pig manures, respectively. The vitality of H9N2 and EMCV significantly declined as a result of composting. However, at the completion of the composting process after 168 h, inactivation effect appeared to be more sensitive in H9N2 than in EMCV, since it occurred within 1 h and 24 h of the start of the composting processes in H9N2 and EMCV, respectively, being more effective under mechanical aeration conditions. Nowadays, high-throughput sequencing technologies have become an indispensable tool to deeply characterize microbiomes from diverse environmental compartments. Using this technology, Gómez-Brandón et al. [3] revealed that the bacterial communities during the vermicomposting of grape marc over a period of 91 days were dominated by *Proteobacteria* and *Bacteriodetes*. They also found that the higher taxonomical diversity and richness were achieved after 14 days followed by significant increases in functional diversity of bacterial communities. Their findings evidenced that the valorization of distilled grape marc via vermicomposting led to a rich end product with higher bacterial diversity and functional attributes, which may be used as suitable plant growth promoter and/or soil organic amendment.

Six articles dealt with the characterization of the soil microbial responses to organic amendment or to contrasting agricultural practices. In this way, Neher et al. [4] assessed the impact of poultry litter compost (PLC) and dairy manure compost (DMC) on microbial communities of soils planted with spinach in the long term. Using metabarcoding, they evidenced that bacteria in the phylum *Bacteriodetes*, and classes *Flavobacteriia* and *Spingobacteriales*, were more abundant in soils amended with PLC than in those amended with DMC or in the unamended ones. However, fungi did not differ among treatments. Tang et al. [5] tested the performance of several plant growth-promoting rhizobacteria (PGPR), belonging to the genera *Paraburkholderia*, *Burkholderia* and *Serratia*, on maize growth, soil nutrient contents and nutrient use efficiency in soils subjected to different regimes of inorganic fertilization and organic amendment. They concluded that the amount of fertilizer applied into soil can be reduced through the combined use of fertilization and PGPR. On the other hand, Kim et al. [6], using 16S rRNA gene metabarcoding, aimed at identifying microbial groups involved in important soil processes as bio-indicators of improved soil health under different cover cropping and tillage practices. Bacteria were found to be more suitable than fungi and archaea for this purpose. Hence, the bacterial genera *Cellulomonas*, *Solirubacter*, *Alererythrobacter* and *Massilia* were found to be potential bio-indicators of enhanced aerobic decomposition, the family *Nitrosomonadaceae* of ammonia oxidation, and the bacterial genera *Nitrospira* and *Reyranella* of nitrite oxidation and nitrate reduction, respectively. Bai et al. [7] compared the effects of long-term and non-long-term fertilization on root-associated soils of walnut trees. This study pointed out that bacterial and fungal communities were negatively affected by long-term fertilization treatments as the result of an over-accumulation of ammonium-nitrogen (NH_4_^+^) and available phosphorus, which led to soil acidification. Meanwhile, soils non-subjected to long-term chemical fertilization resulted in the improvement of microbial communities involved in nutrient mobilization and plant growth.

This Special Issue includes two review articles. That of Lazcano et al. [8] provided a comprehensive overview on the use of organic fertilizers (OFs) for preserving soil health and sustainability. A special focus was given to the effects of OFs, including raw manure and processed materials, such as digestate, compost, vermicompost and biochar on the community structure and function of nitrifying and denitrifying soil microorganisms. Nitrification and denitrification are among the most important soil N transformations within the soil–plant interface, but there is still a lack of information about the underlying mechanisms related to the influence of OFs on these microbial processes and the consequences on nitrous oxide production. Putting the accent on this matter and with the aid of multi-omics approaches, it will be fundamental for the prediction of N_2_O emissions under different scenarios of land use. Finally, Siles et al. [9] provided a thorough review about the use of co-occurrence network approaches as a proxy to gain insights into the interactions among the members of a microbial community and the identification of its keystone members during the processes of composting and anaerobic digestion and following the application of the respective end products (compost and digestate) into soil. Depending on the degree of these interactions and the species involved, the dynamics of these processes may vary with further consequences on the quality of the end product and its potential usefulness as an organic amendment.
